# Reversing Bleeding Associated With Antiplatelet Use: The Role of Tranexamic Acid

**DOI:** 10.7759/cureus.10290

**Published:** 2020-09-07

**Authors:** Kyle Fischer, Fatema Bodalbhai, Elizabeth Awudi, Salim Surani

**Affiliations:** 1 Pharmacy, Texas A&M Irma Lerma Rangel College of Pharmacy, Kingsville, USA; 2 Pharmacy, Texas A&M Irma Lerma Rangel College of Pharmacy, College Station, USA; 3 Pharmacy, Corpus Christi Medical Center, Corpus Christi, USA; 4 Internal Medicine, Corpus Christi Medical Center, Corpus Christi, USA; 5 Internal Medicine, University of North Texas, Dallas, USA

**Keywords:** tranexamic acid, hemorrhage, bleeding, antiplatelet, reversal, clopidogrel

## Abstract

Dual antiplatelet therapy (DAPT) is the mainstay of therapy in patients that have been diagnosed with coronary artery disease. DAPT has known risk factors such as an increased risk of bleeding, and, currently, no specific medication is indicated to reverse bleeding associated with antiplatelet use. One medication that may help reduce blood loss is tranexamic acid (TXA). A retrospective review of the literature regarding TXA in the setting of antiplatelet associated bleeding through a systematic search strategy was conducted. This review of the literature followed the PRISMA (Preferred Reporting Items for Systematic Reviews and Meta-Analysis) guidelines and included seven studies. Multiple studies demonstrated the impact on platelet function resulting from administering TXA through lower volumes of blood loss, lower transfusion requirements, and lower incidence of reoperations. TXA is not widely recommended to reverse antiplatelet medications; however, it is widely available, has a positive track record for use in various types of bleeding, and is relatively inexpensive and safe. Large-scale randomized trials are warranted to make a strong recommendation for TXA in reversing bleeding associated with antiplatelet therapy.

## Introduction and background

Antiplatelet agents such as acetylsalicylic acid (ASA) and clopidogrel are essential in the management of acute coronary syndrome (ACS). However, studies have indicated an increased risk of bleeding with dual antiplatelet therapy (DAPT) [1,2]. Bleeding associated with antiplatelet therapy is a known risk factor that may result in poor outcomes with an increase in morbidity and mortality [3]. Along with poor outcomes, an increase in hospital length of stay, mortality, and cost correlates to bleeding events due to antiplatelet agents [4].

Currently, there is no specific reversal agent that truly counteracts the antiplatelet effect. The most recent Neurocritical Care guidelines suggest a single dose of desmopressin (DDAVP) for intracranial hemorrhages associated with antiplatelet agents such as aspirin and clopidogrel [5]. DDAVP improves platelet function by increasing the release of von Willebrand factor (vWF) and factor VIII from the endothelium [6]. Additionally, one agent that may also provide benefit in antiplatelet associated bleeding is tranexamic acid (TXA). TXA has shown to be a productive agent to halt bleeding in a variety of clinical settings as in trauma, with an excellent safety profile [7,8]. TXA is mainly known for its inhibition of fibrinolysis by preventing the conversion of plasminogen to plasmin [9]. Plasmin causes platelet dysfunction through proteolytic degradation and redistribution of platelet glycoprotein IB and IIB/IIIa receptors [10]. In ACS, inhibiting glycoprotein receptors is common with antiplatelet agents, as they are essential in platelet aggregation. TXA blocks the conversion of plasminogen to plasmin, thus mitigating the effect of plasmin on degrading glycoprotein Ib receptors [11]. Theoretically, TXA will allow the interaction between glycoprotein Ib receptors and vWF, leading to improved platelet function and reduced bleeding risk through its unique mechanism in the setting of antiplatelet bleeding.

Several studies have hinted at the evidence that TXA may improve platelet function in the context of antiplatelet agents. Studies examining coronary artery bypass graft (CABG) patients have provided positive results that indicate TXA improves in vitro platelet function among patients who had received antiplatelet therapy as well as demonstrated a reduction in operative blood loss [10,12]. The promising findings from the literature are trending in the direction where TXA may have an added role in the clinical setting.

Furthermore, balancing ischemic and bleeding risk is an evolving framework where a multidisciplinary approach should be taken to optimize therapy. The purpose of this review was to shed light on a potential therapy option that is widely available to reduce bleeding associated with antiplatelet agents. Currently, TXA is not widely recommended to reverse antiplatelet medications, and this review will provide insight into the role TXA may play.

## Review

Methods

Data Source

This was a retrospective review of the literature on TXA in the setting of antiplatelet associated bleeding through a systematic search strategy. This study followed the guidelines from the Preferred Reporting Items for Systematic Reviews and Meta-Analysis (PRISMA) Statement 2009. It used the PRISMA flow diagram as a template to record the flow of information through the review [13]

Search Strategy

**Figure 1:**
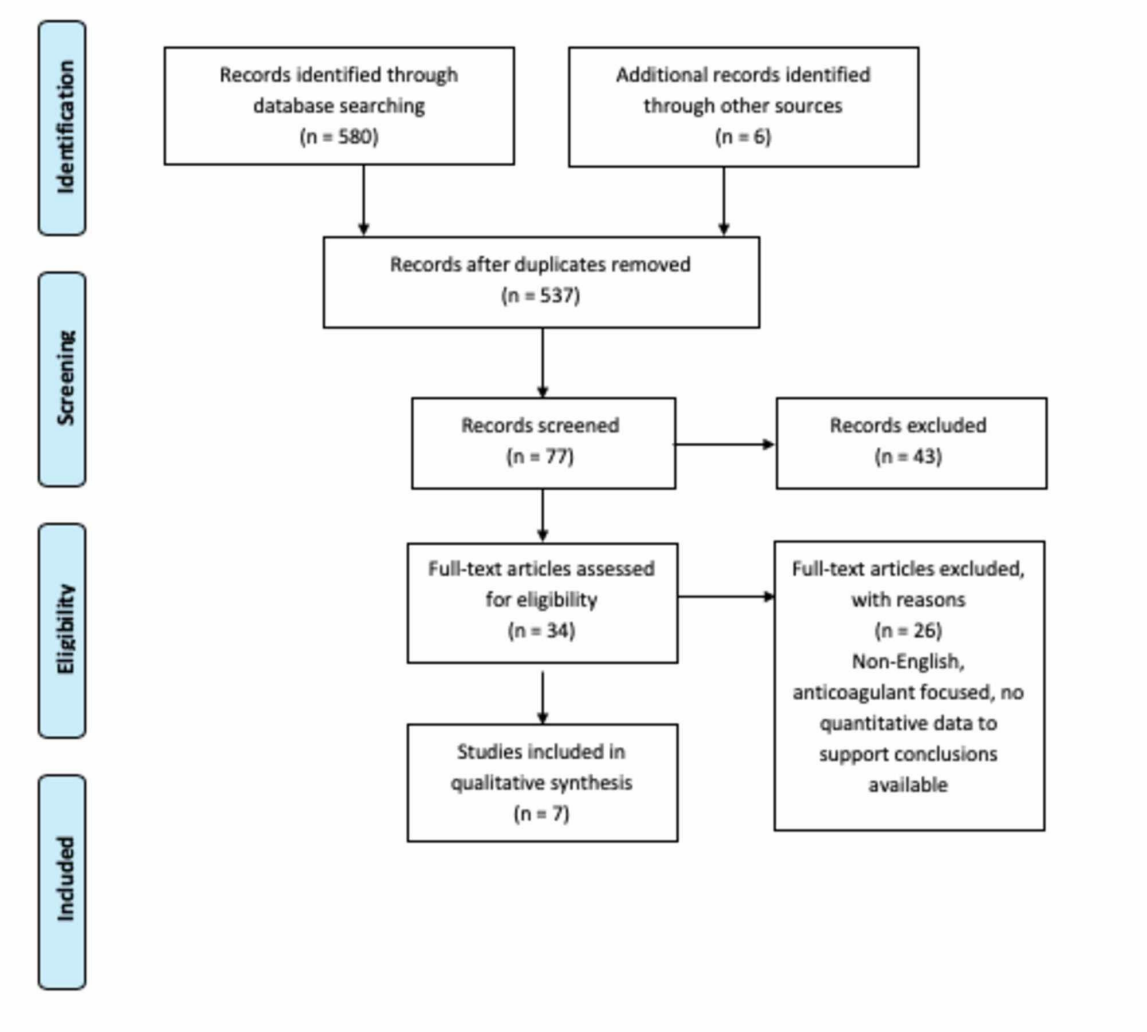
A PRISMA flowchart of the literature and search strategy of studies PRISMA, Preferred Reporting Items for Systematic Reviews and Meta-Analyses

We conducted a systematic literature search of PubMed/MEDLINE databases to identify relevant studies published between 2002 and 2020. The searches were conducted with the keywords and phrases "TXA improving platelet function", "Tranexamic acid AND DAPT", "clopidogrel tranexamic acid", and "reversing antiplatelets TXA." We also screened references from the included studies. The last search concluded on July 31, 2020. The PRISMA flowchart of the literature and search strategy of the studies is shown in Figure 1.

Eligibility Criteria

Retrieved articles were first condensed to remove duplicates using the EndNote program. The pieces that were reduced then were screened in two steps for eligibility defined in the PICOS (population, intervention, comparator, outcomes, and study design) framework in Table 1 [14]. The initial screening was conducted using titles and abstracts. If the study did not meet the inclusion criteria, it was excluded. The second step included retrieving full-text articles and applying the inclusion/exclusion criteria to determine eligibility. The screenings of all articles were performed by two authors (F.B. and K.F.) in an unblinded, standardized manner. Any discrepancies were resolved through discussion or by involving a third and fourth reviewer (E.A. and S.S.) until consensus was reached. Inclusion and exclusion criteria are shown in Table 1.

**Table 1:** Inclusion and exclusion criteria for article selection

Criteria	Inclusion	Exclusion
Population	Patients receiving antiplatelet monotherapy or DAPT that got TXA to improve platelet function	Patients only on anticoagulants, concomitant antifibrinolytics
Intervention	Tranexamic acid	Combination antifibrinolytic therapy used
Comparator	Placebo	N/A
Outcomes	The volume of blood loss, transfusion requirements, the incidence of reoperation due to blood loss	Other than listed under inclusion criteria
Study design	Case studies, observational studies, randomized controlled trials, review articles, opinion/experts articles	Other than listed under inclusion criteria

Data Extraction

Data extraction was performed by two authors (F.B. and K.F.) using a standardized extraction form that included author, year of publication, study design, number of cases/controls, findings, and positives/negatives. The primary outcome measures were blood and platelet transfusion requirements, reoperation due to significant bleeding, and volume of blood loss.

Results 

TXA has been studied in multiple trials as an agent to minimize bleeding in patients receiving antiplatelet monotherapy or DAPT who are undergoing surgery. A systematic review using the inclusion and exclusion criteria outlined in Table 1 was conducted, and seven clinical trials were identified.

The use of TXA in all seven trials resulted in an observable decrease in the identified primary outcomes of blood loss, reoperations, and blood/platelet transfusion requirements [11,15-20]. There was a statistically significant decrease in blood/platelet transfusion requirements (as represented by a p-value less than or equal to 0.05) in three of the six trials that included transfusion requirements as a study outcome [12,15-20]. A statistically significant decrease in the volume of blood loss was present in all three of the trials that quantified the volume of blood loss in their results [12,16,18]. The incidence of reoperation due to bleeding was deemed statistically significant in two of the three trials, where it was reported as an outcome [16,17,20].

A noteworthy result from the indicated trials is the impact of time on the benefits of TXA therapy. Weber et al. concluded that no significant changes in primary outcomes existed between study groups when patients had ceased antiplatelet treatment at least seven days before surgery (p = 0.294) [10]. This was countered by Shi et al., which resulted in a benefit from TXA administration regardless of antiplatelet cessation time [16]. In the two studies that measured the primary outcome of blood loss after a certain period postoperative, both studies concluded that there was no significant difference between groups (p = 0.66 and p = 0.720) [12,15]. Table 2 summarizes these clinical trials by study design, number of cases, and their findings.

**Table 2:** Studies on TXA reversing antiplatelet agent induced bleeding ADP, adenosine diphosphate; AU, aggregation units; CI, confidence interval; DAPT, dual antiplatelet therapy; MBC, major bleeding complications; PH, pocket hematoma; pRBC, packed red blood cell; RBC, red blood cell; RCT, randomized control trial; RR, relative risk; SD, standard deviation; TXA, tranexamic acid

Authors	Study Design	Number of Cases/Controls	TXA Dosing	Positive/Negative Findings
Pleym et al., [18]	Prospective, randomized, double-blind, placebo-controlled, parallel-group trial	TXA group (n = 40) and placebo (n = 39)	30 mg/kg	The TXA group had significantly less postoperative bleeding compared to placebo (mean (SD): 475 (269) vs 713 (243) mL; p< 0.001). The placebo group had significantly more volume of blood transfused (566 (274) vs 356 (280) mL; p=0.001).
Weber et al. [10]	Prospective, observational study	20 patients having ceased antiplatelet therapy 7 days before surgery; 20 patients on antiplatelet therapy up until the day before surgery. All patients received TXA, and blood tests were conducted before and after the first TXA dose.	Cumulative dosage of 6 grams	Statistically significant increase in arachidonic acid-induced (295 (280/470) AU*min vs. 214 (83/409) AU*min; p = 0.01) and ADP-induced platelet aggregation (560 (400/760) AU*min vs. 470 (282/550) AU*min; p = 0.013) after receiving TXA selectively in patients who did not cease DAPT before surgery. No significant changes in platelet aggregation were observed post-TXA in the group that ceased DAPT (p = 0.294).
Ahn et al. [15]	RCT, double-blind, placebo-controlled	Control group (n = 38) and TXA group (n = 38)	1 gram bolus followed by 200 mg/h	Patients in the TXA group received a significantly smaller amount of pRBC transfusions compared to control, though blood loss was lower in the TXA group for the first 4 hours after the operation and similar between both groups after 4 hours. Fewer patients required perioperative pRBC transfusions in the TXA group (20 vs. 27; p = 0.098) Fewer patients in the TXA group required pRBC than the control group (p = 0.066).
Shi et al. [16]	Multicenter, randomized, blind trial	TXA group (n = 274) and placebo group (n = 278)	10 mg/kg bolus and 10 mg/kg maintenance dose	TXA reduced blood loss compared to placebo (p< 0.001). TXA reduced major bleeding (43.8% vs. 63.3%; p< 0.001; RR: 0.69; 95% CI: 0.59-0.81). TXA reduced volume of RBCs transfused (p< 0.001). TXA reduced transfusion exposure (60.9% vs 79.5%; p< 0.001; RR: 0.76; 95% CI: 0.68-0.85). The overall incidence of reoperation for bleeding was cut down from 6.83% to 1.82% in the TXA group (p = 0.004; RR: 0.27; 95% CI: 0.10-0.71). TXA showed benefit regardless of preoperative clopidogrel and its cessation time.
Beton et al. [17]	Retrospective analysis	TXA group (n = 16) and control group (n = 21)	500 mg/5 mL	PH occurred in 7.7% of patients in the TXA group and 26.5% patients in the control group (p = 0.013). MBC was reported in 5.8% of patients in the TXA group and 20.5% patients in the control group (p = 0.024).
Myles et al. [20]	Multicenter, double-blind trial	TXA group (n = 2311) and placebo group (n = 2320)	758 patients received 100 mg/kg and then protocol changed, with 1553 patients receiving 50 mg/kg	Total units of blood products transfused were 4331 in the TXA group and 7994 in the placebo group, which was 46% fewer units in the TXA group (p < 0.001). Major hemorrhage leading to reoperation was 1.4% of patients in the TXA group and 2.8% of patients in the placebo group (p = 0.001).
Banihashem et al. [12]	Prospective, randomized, double-blind	TXA group (n = 60) ad control group (n = 60)	10 mg/kg	Slightly higher platelet counts were observed in the TXA group compared to placebo. The average volume of blood loss for patients with clopidogrel exposure within 48 hours before surgery was lower for the TXA group (p = 0.03). There was no statistically significant difference in the volume of postoperative bleeding between groups at 48 hours (p = 0.66). Perioperative transfusion requirements of platelets of pRBC were similar between both groups (p = 0.66).

Discussion

To summarize the current literature regarding bleeding associated with antiplatelet use, this review identified and analyzed the role TXA may play in this setting. Stemming back from 2003, Pleym et al. showed that administering TXA before cardiopulmonary bypass in CABG patients significantly reduced postoperative bleeding in patients treated with aspirin before surgery. A smaller volume of blood loss was also reported for the TXA group. In regard to transfusion requirements, the study was not adequately powered to detect a difference, but the authors noted a smaller number of packed red blood cells needed in the TXA group [18]. A decade later, Shi et al. assessed the protective effects of TXA on clopidogrel in CABG patients. Shi et al. looked at clopidogrel, unlike Pleym et al., who looked at aspirin. The findings from Shi et al. were consistent from the previous study in that TXA reduced the risk and exhibited protective qualities in patients with clopidogrel exposure within seven days before CABG [16]. Myles et al. also assessed patients who underwent a CABG surgery while on aspirin. The authors concluded that TXA lowered the risk of bleeding compared to placebo without increasing the risk of death or thrombotic complications within 30 days after surgery [19,20].

In 2011, Weber et al. set out to analyze the efficacy of TXA to improve platelet function in the setting of DAPT. DAPT is a mainstay of treatment therapy for patients with various cardiovascular diseases, and bleeding is a significant risk factor [21]. Antiplatelet agents are known to cause platelet dysfunction, and Weber et al. showed that TXA partially reversed platelet aggregation dysfunction that was caused by antiplatelet therapy [10]. One year later, Ahn et al. evaluated TXA on blood loss and transfusion requirements in preoperatively anemic patients who continued DAPT within five days of receiving a CABG. The results were positive as TXA reduced the amount of packed red blood cells and transfusion requirements compared to the placebo [15]. Bleeding complications during cardiac electronic device implantation in patients receiving antiplatelet therapy have been a challenge to combat for some time. Beton et al. performed a retrospective analysis of patients undergoing cardiac electronic device implantation while receiving DAPT or warfarin therapy. Beton et al. found that topical TXA application reduced the incidence of pocket hematomas and major bleeding complications [17]. This finding was also consistent with a systematic review article published in 2013 that highlighted the efficacy of topical TXA in reducing bleeding and blood transfusions [22]. The latest study examining the effects of TXA in preventing bleeding in the setting of antiplatelet agents was conducted in 2019 by Banihashem et al. This was a prospective randomized, double-blind clinical trial that assessed if TXA prevented blood loss in patients on clopidogrel and aspirin within five days of undergoing a CABG. Banihashem et al. inferred that blood transfusion, although not significantly different between TXA and placebo, was less in the TXA group. Average blood loss in the TXA group was substantially lower, and it was concluded that TXA should be used before surgery in all patients to prevent blood loss due to antiplatelet agents [12]. The pharmacodynamics and pharmacokinetics are listed in Table 3.

**Table 3:** PD and PK of antiplatelet reversal agents DDAVP, desmopressin; IV, PD, pharmacodynamics; PK, pharmacokinetics; intravenous; TXA, tranexamic acid

Medication	Dosing	PD/PK	Common Adverse Effects
TXA [23]	1 gram loading dose IV push over 10 minutes 1 gram IV as a gradual infusion over 8 hours for the maintenance dose	Half-life: around 2 hours; excretion: urine	Abdominal pain, hypotension
DDAVP [24]	0.4 mcg/kg x 1 dose	Half-life: around 30 minutes; excretion: urine	Hyponatremia, Hypertension

Although there is currently no specific reversal agent that offsets the antiplatelet effect, DDAVP is arguably the agent of choice to improve platelet function regarding bleeding associated with antiplatelet use [5]. There are still many questions and concerns about what agents may be used to counteract bleeding associated with antiplatelet use, and through the review of the literature conducted, we explored a commonly used and widely available agent for limiting bleeding. TXA may help fill this clinical gap as it works to prevent the binding of plasminogen to fibrin and preserve platelet function [9]. The evidence presented in this review is of fair quality and has been consistent in showing the possible benefits TXA may exert throughout the studies. One thing to consider when analyzing the different studies is the diversity of dosing for TXA. Careful consideration of dosing and administration within a multidisciplinary team is warranted to ensure optimal outcomes. The Neurocritical Care Society and the Society of Critical Care Medicine recommend a dose of TXA for intracranial hemorrhage associated with thrombolytic treatment to be 1 gram (or 10 to 15 milligrams per kilogram) once administered at a rate not exceeding 100 milligrams per minute [5]. This recommendation may be extrapolated in the setting of antiplatelet associated bleeding.

Moreover, given the emerging body of evidence, we have presented several studies suggesting that TXA may be a potential therapeutic option that could improve platelet function in the context of antiplatelet agents. The use of TXA is not widely recommended to reverse antiplatelet agents; however, TXA is widely available, has proven to be safe, tolerable, and efficacious in various types of bleeding, and is relatively inexpensive.

Some limitations of this review should be considered. First, this review is retrospective in nature and is susceptible to bias in data selection. Secondly, the trials are diverse regarding dosing TXA, and a definitive conclusion is not suitable. Large-scale randomized trials are needed to make a firm recommendation encompassing safety and efficacy for TXA in the role of reversing bleeding associated with antiplatelet therapy.

## Conclusions

TXA is a viable option that may decrease bleeding complications arising from either antiplatelet monotherapy or DAPT by improving platelet function. Multiple studies demonstrated the impact on platelet function resulting from administering TXA through lower volumes of blood loss, lower transfusion requirements, and lower incidence of reoperations. These benefits were observed in both patients who continued antiplatelet therapy up until surgery and those who ceased antiplatelet treatment a week before surgery.
